# Dynamics of neutralizing antibody responses to SARS-CoV-2 in patients with COVID-19: an observational study

**DOI:** 10.1038/s41392-021-00611-6

**Published:** 2021-05-18

**Authors:** Xin Xu, Sheng Nie, Yanqun Wang, Quanxin Long, Hong Zhu, Xiaoyong Zhang, Jian Sun, Qinglang Zeng, Jincun Zhao, Li Liu, Ling Li, Ailong Huang, Jinlin Hou, Fan Fan Hou

**Affiliations:** 1 State Key Laboratory of Organ Failure Research, National Clinical Research Center for Kidney Disease, Guangdong Provincial Clinical Research Center for Kidney Disease, Nanfang Hospital, Southern Medical University, Guangzhou, China; 2 State Key Laboratory of Respiratory Disease, National Clinical Research Center for Respiratory Disease, Guangzhou Institute of Respiratory Health, the First Affiliated Hospital of Guangzhou Medical University, Guangzhou, China; 3 Key Laboratory of Molecular Biology on Infectious Diseases, Ministry of Education, Chongqing Medical University, Chongqing, China; 4 The People’s Hospital of Honghu, Honghu, China; 5 Kingmed Medical Laboratory, Guangzhou, China

**Keywords:** Infectious diseases, Infectious diseases

## Abstract

Our understanding of the protective immunity, particularly the long-term dynamics of neutralizing antibody (NAbs) response to SARS-CoV-2, is currently limited. We enrolled a cohort of 545 COVID-19 patients from Hubei, China, who were followed up up to 7 months, and determined the dynamics of NAbs to SARS-CoV-2 by using a surrogate virus neutralization test (sVNT). In our validation study, sVNT IC_50_ titers and the neutralization rate measured at a single dilution (1:20) were well correlated with FRNT titers (*r* = 0.85 and 0.84, respectively). The median time to seroconversion of NAbs was 5.5 days post onset of symptoms. The rate of positive sVNT was 52% in the first week, reached 100% in the third week, and remained above 97% till 6 months post onset. Quantitatively, NAbs peaked in the fourth week and only a quarter of patients had an estimated peak titer of >1000. NAbs declined with a half-time of 61 days (95% CI: 49–80 days) within the first two months, and the decay deaccelerated to a half-time of 104 days (95% CI: 86–130 days) afterward. The peak levels of NAbs were positively associated with severity of COVID-19 and age, while negatively associated with serum albumin levels. The observation that the low-moderate peak neutralizing activity and fast decay of NAbs in most naturally infected individuals called for caution in evaluating the feasibility of antibody-based therapy and vaccine durability. NAbs response positively correlated with disease severity, warning for the possibility of repeat infection in patients with mild COVID-19.

## Introduction

The entry of SARS-CoV-2 into its target cells depends on binding between the receptor-binding domain (RBD) of the viral spike protein and its cellular receptor, angiotensin-converting enzyme 2 (ACE2).^1–3^ The spike protein is highly immunogenic and the target of many neutralizing antibodies (NAbs).^4^ It has been demonstrated that convalescent blood samples obtained from individuals who recover from COVID-19 contain neutralizing activity,^5^ suggesting that humans are intrinsically capable of generating antibodies that potently neutralize SARS-CoV-2. Thus, understanding neutralizing responses, especially NAbs to RBD of SARS-CoV-2, is essential in determining the onset of humoral immunity, evaluating potential capacity of viral clearance, identifying donors for convalescent plasma therapy, and assessing vaccine efficacy during clinical trials or after large-scale vaccination.

However, our understanding of the protective immunity to SARS-CoV-2 is currently limited. Little is known about the dynamics of RBD-targeting NAbs, including its generation, longevity, and correlation with the clinical settings, particularly at population level.

Lack of reliable and versatile tests for assessment of neutralizing activity remains a big challenge. The conventional virus neutralization tests, such as focus reduction neutralization test (FRNT) and pseudo-virus-based neutralization test remain the only platforms for detection of NAbs. Both tests require live viruses and cells, and days to obtain results, and thus unsuitable for large-scale measurement and population study.

Recently, a surrogate virus neutralization test (sVNT) has been established based on antibody-mediated blockage of ACE2-spike protein-protein interaction.^6^ sVNT quantifies RBD-targeting NAbs without the need for biosafety level 3 containment or any live virus or cells. Here, by using this sVNT, we assessed the dynamics of RBD-targeting NAbs response to SARS-CoV-2 in a cohort of 545 COVID-19 patients up to 7 months post onset of symptoms, and explored the clinical correlates to the peak NAbs response in individuals.

## Results

Our study cohort included 545 COVID-19 patients who were diagnosed between January and March 2020 and managed at the designated hospitals in Honghu and Jingzhou, Hubei province. All cases were confirmed by RT-PCR testing. Of 545 patients, the mean age was 50.9 years, 286 (52.5%) were males, 54 (9.9%) were severe, and 379 (69.5%) had at least two time points of sera collection during the course of study. The 25^th^, 50^th^, and 75^th^ percentiles of follow-up time were 45 days, 62 days, and 175 days, respectively, post onset. The flowchart of sample selection was presented in Supplementary Fig. 1. The characteristics of the cohort and selected datasets are summarized in Table 1.

**Table 1:** Characteristics of the COVID-19 patients

	*Total population* *n* = *545*	*Peak analysis* *n* = *328*	*Decay analysis* *n* = *194*	*6-month visit* *n* = *197*
*Age, year, Mean ± sd*	50.9 ± 15.0	49.9 ± 14.5	52.2 ± 13.9	55.9 ± 13.3
*Age, N(%)*				
18 ~ 40 year	140 (25.7%)	88 (26.8%)	41 (21.1%)	28 (14.2%)
41 ~ 60 year	262 (48.1%)	159 (48.5%)	98 (50.5%)	93 (47.2%)
61 ~ 90 year	143 (26.2%)	81 (24.7%)	55 (28.4%)	76 (38.6%)
*Gender, N (%)*				
female	259 (47.5%)	155 (47.3%)	86 (44.3%)	92 (46.7%)
male	286 (52.5%)	173 (52.7%)	108 (55.7%)	105 (53.3%)
*Disease severity*^*a*^*, N (%)*				
mild	491 (90.1%)	304 (92.7%)	181 (93.3%)	181 (91.9%)
severe	54 (9.9%)	24 (7.3%)	13 (6.7%)	16 (8.1%)
*Number of antibody testing, N (%)*				
1	166 (30.5%)	26 (7.9%)	0 (0%)	26 (13.2%)
2	111 (20.4%)	76 (23.2%)	31 (16.0%)	24 (12.2%)
3	144 (26.4%)	126 (38.4%)	87 (44.8%)	76 (38.6%)
≥4	124 (22.8%)	100 (30.5%)	76 (39.2%)	71 (36.0%)
*Number of RT-PCR testing, Mean ± sd*	7.0 ± 2.3	7.3 ± 2.2	7.2 ± 2.1	6.9 ± 2.1
*Days from symptom to PCR positive*^*b*^*, Mean ± sd*	11.7 ± 8.4	12.6 ± 8.1	13.1 ± 8.1	12.3 ± 8.0
*Duration of COVID-19*^*c*^*, day, Mean ± sd*	25.6 ± 13.1	26.8 ± 12.6	29.8 ± 12.4	28.7 ± 13.2
*Length of follow up*^*d*^*, days*	62 (45, 175)	63 (49, 179)	174 (62, 183)	180 (174, 184)
*Use of hydroxychloroquine*^*e*^*, N (%)*	49 (13.5%)	39 (14.1%)	22 (12.8%)	21 (15.1%)
*Use of glucocorticoids*^*e*^*, N (%)*	178 (48.9%)	135 (48.9%)	90 (52.3%)	79 (56.8%)

^a^ Severe COVID-19 was defined as need for ICU, respiratory failure, mechanical ventilation, or death.

^b^ in symptomatic patients. Symptoms considered included fever, coughing, sore throat, tiredness, dyspnea, aches and pains, diarrhea, and loss of taste or smell.

^c^ defined as days from onset of symptoms to virus shedding (RT-PCR test turning from positive to negative).

^d^ presented as median (25^th^, 75^th^ percentile).

^e^ in patients with medical information.

### Validation of a sVNT

We performed validation study on sVNT to address the following three questions: (1) what is the specificity and sensitivity of this assay measured in 1:20 diluted sera? (2) could sVNT titer (IC_50_) serve as a surrogate for the titer (IC_50_) obtained from FRNT? and (3) could measurement from sVNT at a single dilution (1:20) or total binging IgG level serve as a surrogate for FRNT titer?

First, we evaluated the specificity and sensitivity of sVNT when measuring neutralization rate in 1:20 diluted sera (NR_20_) using sera from 194 healthy individuals as negative controls, and sera from 395 COVID-19 patients collected on days 21–60 post onset of symptoms as positive controls, resulting in a specificity of 100% (95% confidence interval: 98.1–100%) and a sensitivity of 97.2% (95% confidence interval: 95.1–98.4%) when using a cutoff value of 20% for NR_20_.

**Fig. 1:**
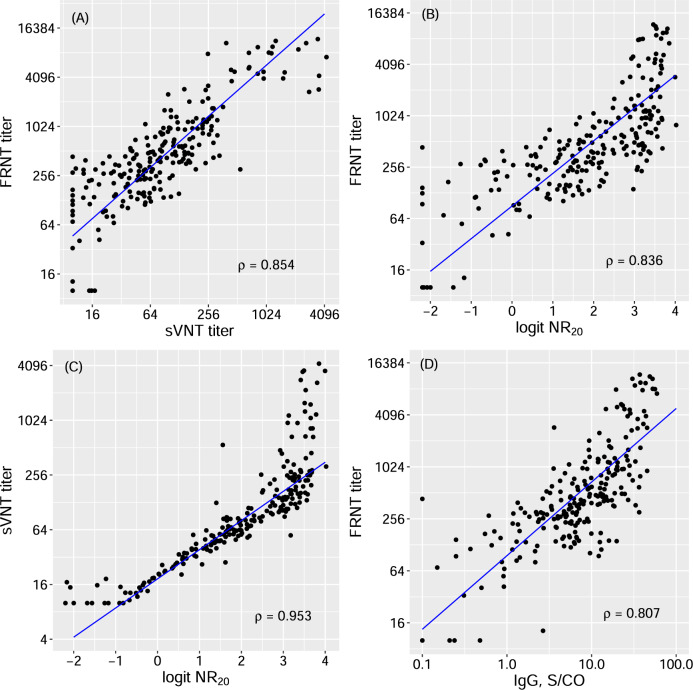
Correlations among different measurements of NAbs and IgG against SARS-CoV-2. **A** NAbs IC_50_ titer by focus reduction neutralization test (FRNT) vs NAbs IC_50_ titer by surrogate virus neutralization test (sVNT); **B** NAbs IC_50_ titer by FRNT vs logit of sVNT neutralization rate at 1:20 dilution of sera (logit NR_20_); **C** NAbs IC_50_ titer by sVNT vs logit NR_20_; **D** NAbs IC_50_ titer by FRNT vs the level of total binding IgG. The blue lines are the regression lines between two measurements estimated by Deming regression. *ρ* Spearman’s correlation coefficients, S/CO ratio of the chemiluminescence signal over the cutoff value

Next, we compared the titers from FRNT and sVNT measured in 246 convalescent sera within 6 months post onset of symptoms (Supplementary Fig. 2). The titers from sVNT were significantly correlated with FRNT titers, with a Spearman’s correlation coefficient of 0.85 (Fig. 1A), suggesting that it could be a good surrogate for FRNT titers at a relative scale.

We then evaluated the relationship of antibody (RBD-targeting NAbs and total binding IgG) levels measured at 1:20 sample dilution with FRNT titers. The logit transform of NR_20_, i.e., log [NR_20_/(1- NR_20_)], had a log-linear relationship with both FRNT and sVNT titers, with correlation coefficients of 0.84 and 0.95, respectively (Figs. 1B and 1C). In comparison, the correlation between total binding IgG level and FRNT titer was slightly lower at 0.81 (Fig. 1D). The fact that logit NR_20_ had a good log-linear relationship with FRNT titers and that NR_20_ was much easier and faster to measure make logit NR_20_ a suitable surrogate for log FRNT titers. In our regression analysis of the validation set, the relationship between FRNT titers and logit NR_20_ was estimated as log (FRNT titer) = 4.5 + 0.88 × logit (NR_20_) (Equantion 1), where log denotes the natural logarithm. Logit NR_20_ values of 0.12, 1.38, and 2.74 were equivalent to a FRNT titer of 100, 300, and 1000, respectively. As a result, we chose to use logit NR_20_ as a surrogate for log titers of NAbs in subsequent studies.

### Dynamic response of neutralizing antibody in COVID-19 patients

**Fig. 2:**
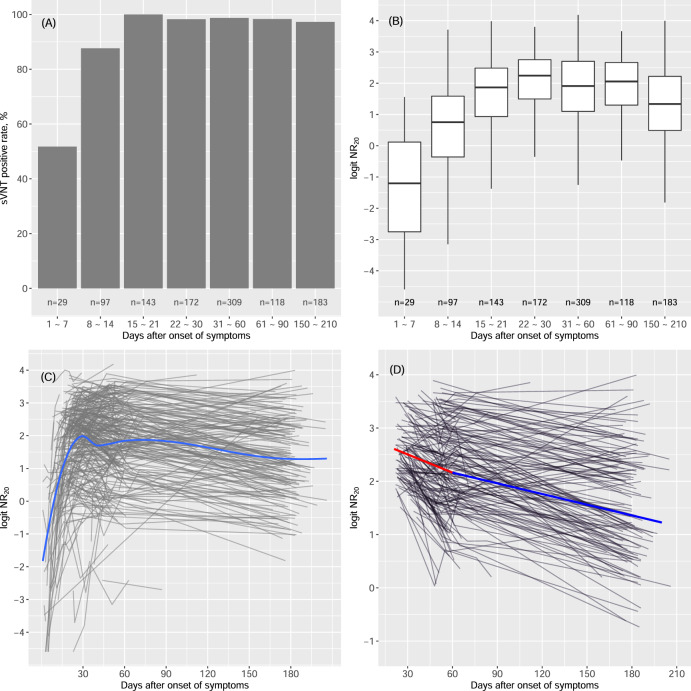
Dynamics of NAbs response to SARS-CoV-2. **A** The positive rate of NAbs (measured by sVNT) stratified by time post onset. A NR_20_ value (neutralization rate at 1:20 sample dilution) of ≥20% was regarded positive. **B** Boxplot of the titers of NAbs stratified by time post onset. **C** The trajectories of NAbs in patients. Each gray line represents the trajectory of a patient. The blue line is the loess smooth curve of the trajectories. **D** Decline of NAbs over time in patients with peak logit NR_20_ > 1.3 (25^th^ percentile of peak distribution). Each gray line represents the post-peak trajectory of a patient. The blue line is the loess smooth curve of the trajectories

We performed survival analysis to estimate the median time to seroconversion in 317 symptomatic COVID-19 patients who had at least one antibody measurement within the first 30 days post onset (Supplementary Fig. 3). Seroconversion of RBD-targeting NAbs and total binding IgG occurred on average one day early than IgM. The median time of seroconversion (95% confidence interval) was 5.5 (2–6), 5.5 (3–6), and 6.5 (3–8.5) days post onset of symptoms for NAb, IgG, and IgM, respectively. The rate of positive sVNT was 52% in the first week post onset, reached 100% in the third week, and remained above 97% till 6 months post onset (Fig. 2A). Quantitatively, neutralizing antibody peaked in the fourth week with a median logit NR_20_ of 2.2 (equivalent to a FRNT titer of 624, see Equation 1), and slowly declined afterward (Figs. 2B–C). The dynamic response of IgG was very similar to that of RBD-targeting NAbs (Supplementary Figs. 4A–B). Compared with IgG and NAbs, IgM tended to have a lower peak and a lower positive rate and a much faster declining after peak. At 6 months post onset, only 42% of sera remained positive for IgM testing.

We calculated the peak titer of RBD-targeting NAbs in a given individual as the highest titer observed between 21^st^ and 60^th^ day post onset. Of 395 patients with an observed peak titer, the median (25^th^ to 75^th^ percentile) of peak logit NR_20_ was 2.2 (1.3 to 2.8), which was equivalent to a FRNT titer of 624 (283, 1058), respectively, indicating that low-moderate levels of NAbs were observed in 75% of patients in this cohort.

It is important to know whether the body can maintain a high activity of NAbs to SARS-CoV-2 after an extended length of time. We used linear mixed-effect models to estimate the post-peak declining rate of NAbs as well as total binding IgG and IgM in 194 patients who had a reasonably high peak titer (logit NR_20_ > 1.3, the 25^th^ percentile) and at least one additional antibody measurement after the peak (Fig. 2D and Supplementary Fig. 4D). NAbs appeared to decline at a faster rate within the first 2 months post onset, in younger individuals and in those with a high level of peak titer (Table 2). The decline rates were similar between genders and strata of severity. Overall, NAbs declined with a half-time of 61 days (95% CI: 49 to 80 days) within the first two months post onset and 104 days (95% CI: 86 to 130 days) afterward. Unlike NAbs, the declining rates of IgG and IgM were not time-dependent. The decay half-time of IgG and IgM was 95 days (95% CI: 85 to 108 days) and 42 days (95% CI: 39 to 47 days), respectively.

**Table 2:** Slopes of decline in logit NR_20_

Slope, *per 10 day*	*coefficient*	*95% confidence interval*	*p-value for interaction*
*Days post onset*			2 × 10^−4^
>60 days	−0.0735	−0.100 to −0.047	
≤60 days	−0.132	−0.168 to −0.096	
*Age*			0.041
>60 years	−0.106	−0.147 to −0.064	
≤60 years	−0.132	−0.168 to −0.096	
*Peak logit NAb*			0.034
>2.5	−0.158	−0.193 to −0.121	
≤2.5	−0.132	−0.168 to −0.096	
*Gender*			0.64
male	−0.126	−0.161 to −0.092	
female	−0.132	−0.168 to −0.096	

Slope of decline in logit NR_20_ was estimated using a linear mixed effect model with random slope and random intercept. Modification of the slope by time window (≤60 days, >60 days post onset), age (≤60, >60 years), peak logit NR_**20**_ level (≤2.5, >2.5), and gender (female, male) was evaluated by adding interaction terms in the model.

We measured FRNT titers (dilution at IC_50_) in 197 COVID-19 patients who participated in the 6-month visit. The follow-up time ranged from 144 to 206 days post onset, with a median of 180 days. The distribution of the titers was presented in Supplementary Fig. 5. Of 197 samples, the highest titer was 3959 and the geometric mean was 358 (95% CI: 308 to 416). Twenty nine (14.7%) had a titer above 1000, while 154 (78.2%) between 100 and 999, and 14 (7.1%) below 100, respectively.

### Clinical correlates of peak response of NAbs

Among 395 COVID-19 patients with an observed peak level of NAbs, 328 had detailed medical information (Table 1 and Supplementary Table 1) and were used for exploring the association between the clinical features of COVID-19 and the peak level of NAbs (logit NR_20_). A total of 57 demographic and clinical features were analyzed, including age, gender, disease severity and duration of COVID-19 (days from onset to the earliest-time RT-PCR testing turning from positive to negative), presence of symptoms, imaging signs of lung infection, comorbidities, complete blood count, and laboratory biochemistry tests (Supplementary List 1). We performed stepwise linear regression analysis of peak logit NR_20_ starting with all 57 variables. The final model from the stepwise regression analysis selected 4 variables (multiple R^2^, 0.22) and is summarized in Table 3. In the model, imaging manifestation of lung infection was the most significant factor. The presence of imaging signs of lung infection was associated with a 0.9 log unit (95% confidence interval: 0.52, 1.27) increase in peak logit NR_20_ compared with that without imaging signs. Age and duration of COVID-19 were also positively associated with peak NAbs, while serum albumin level was negatively associated with peak NAbs (Supplementary Fig. 6).

**Table 3:** Final model of peak logit *NR*_20_ selected by stepwise regression analysis in 328 COVID-19 patients

Variables	coefficients	95% confidence interval	*p*-value
Imaging signs of lung infection, no	reference	–	–
Imaging signs of lung infection, yes	0.852	0.462 to 1.242	2 × 10^−5^
Duration of COVID-19^a^, 10 days	0.163	0.065 to 0.262	0.001
Age, 10 year	0.150	0.061 to 0.240	0.001
Albumin, 10 g/L	−0.392	−0.625 to −0.159	0.001

^a^Defined as days from onset of symptoms to virus shedding (RT-PCR test turning from positive to negative). The multiple R^2^ of the final model was 0.22.

## Discussion

### Main findings

RBD-based sVNT used in this study has been demonstrated to be a robust assay for quantification of RBD-targeting NAbs. Although antibodies to other regions of the S protein can also play a role in virus neutralization,^7^ recent study indicates that the RBD-targeting NAbs are immunodominant during COVID-19.^8^ In this study, we validated sVNT using serum samples from 246 COVID-19 patients within 6 months post onset of symptom. We found an excellent correlation between the IC_50_ titers from FRNT and sVNT. In fact, both NR_20_ and level of total binding IgG were correlated well with FRNT titers, with a correlation coefficient of 0.84 and 0.81, respectively, suggesting that both are suitable surrogates for FRNT in assessing the SARS-CoV-2-specific neutralizing activity.

In this study, we described for the first time the 6-month dynamics of the NAbs response to SARS-CoV-2 in a cohort of COVID-19 patients. Consistent with the previous reports, seroconversion is generally completed within 2 weeks.^9^ In our study, half of the patients seroconverted in the first week, and the seroconversion rate reached 100% in the third week. Neutralizing response peaked at the fourth week with an estimated median peak titer of 624 (25^th^ and 75^th^ percentile: 283, 1058).

Studies on the long-term evolution of antibodies to SARS-CoV-2 remain limited. It is still unclear how long the virus-specific antibodies may persist after infection with SARS-CoV-2. In our study cohort, at 6 months post onset, 97% were still tested positive for NAbs, though on average, the level of NAbs was dropped by one natural log unit (i.e., 63%) from the peak. In the first two months post onset, NAbs declined with a half-time of 61 days. After 2 months, it deaccelerated to a half-time of 104 days. A recent study in 30 COVID-19 patients found that NAbs titers declined by 34.8% within 3 months post onset, which was consistent with our observation. Decline of IgG appeared to be time-independent, with a decay half-time of 95 days (95% CI: 85–108 days). Recently, Ibarrondo *et al* reported a faster decay of SARS-CoV-2-IgG with a half-time of 36 days over 3 months in 31 patients with mild COVID-19.^10^ Several reasons may explain this discrepancy. First, the study populations differ. Ibarrondo’s study only included mild patients, while our study enrolled patients with various clinical settings. For example, our cohort was on average 7 years older. We have shown that younger age is associated with faster decay of antibodies. Second, we used linear mixed-effect model with random slope and random intercept to estimate the slope, while Ibarrondo’s study used simple linear regression. Third, existence of ethnic difference in the decline rate could not be excluded. The trend in RBD-targeting NAbs dynamics was similar to that of IgG antibody observed in the same cohort and in the previous report.^9^ NAbs targeting RBD are likely the crucial component of the protective immunity against SARS-CoV-2.^11,12^ The observed fast decay of NAbs in the most naturally infected individuals calls for caution in evaluating the feasibility of antibody-based therapy for COVID-19 and vaccine durability.

The availability of detailed medical information of the study cohort enabled us to identify clinical settings that are mostly associated with the peak NAbs. Unexpectedly, we found that disease severity, indicated by obvious symptoms, pulmonary lesions, and longer virus shedding time, was positively associated with peak NAbs response. In addition, age was positively correlated with the peak-neutralizing activity, while the level of serum albumin was negatively correlated. These may also be explained by the association between disease severity and the NAbs response since both patients with old age and low albumin are more susceptible to severe disease. Our findings were consistent with a recent report that severity was positivity correlated with NAbs response.^13^ These results sound a warning for the possibility of repeat infection in patients with mild COVID-19.

### Strengths and limitations

A strength of our study was the large sample size, including patients with various clinical settings and longitudinal tracking of NAbs up to 7 months, making it feasible to accurately estimate the dynamics of NAbs response and compare the responses among clinic subgroups. In addition, few new COVID-19 cases were reported in Hubei province since May, making it very unlikely that our analysis of long-term NAbs response was confounded by re-infection. This study also has limitations. The longest follow-up time was 7 months. Longer follow-up study is required to assess the long-term dynamics of NAbs after this period. Our cohort was exclusively Chinese. Whether the findings from our study are generalizable to other populations requires further validation.

In summary, we have determined the dynamics of RBD-targeting NAbs response to SARS-CoV-2 up to 7 months post onset in a large cohort of COVID-19 patients. Most patients have low-moderate peak-neutralizing activity. NAbs declines quickly in the first two months post onset, and the decline deaccelerates afterward with a half-time of 104 days. NAbs response positively correlated with disease severity. Our data have thus provided important insights for humoral protective immunity against SARS-CoV-2.

## Methods

### Study population

The study cohort consisted of all COVID-19 patients (*n* = 545) who had been diagnosed between January and March and managed at five local hospitals designated for the care and management of COVID-19 patients in Honghu and Jingzhou, Hubei province. All cases were confirmed by RT-PCR testing. According to the Health Commission of Hubei province, until end of March, a total of 1580 COVID-19 cases had been reported from Honghu and Jingzhou of Hubei province. So, our cohort represented 34% of the COVID-19 cases in the region. Demographic and clinical information, including gender, age, history of diseases, and presence of symptoms at onset, were extracted from the electronic medical records. Reports of chest imaging examination and laboratory tests, including SASR-CoV-2 RT-PCR testing, were exported from the hospital imaging and laboratory information systems. Under the regulation for COVID-19 management, these patients were isolated and managed at the designated hospitals until viral shedding. After discharge, patients were followed up biweekly until 60 days post onset. In late July, we conducted another round of follow-up in which 197 convalescent patients participated. Serum samples were collected in local hospitals. All samples were inactivated at 56 °C for 30 min and stored at −80 °C before use. In addition, we have collected sera from 194 healthy individuals who took regular physical checkup at Nanfang hospital in May and were tested negative for both RT-PCT testing and total binding IgG and IgM assay. Sera from these individuals were used as negative controls in the validation study. The flowchart of sample selection was presented in Supplementary Fig. 1. The Medical Ethics Committee of Nanfang Hospital approved the study. Written consents were obtained from all study participants.

### SARS-CoV-2 sVNT and calculation of IC_50_

While FRNT remains the gold standard for serological testing and determining immune protection, it is not practical for large scale epidemiological studies due to its low throughput and stringent biosafety requirement. sVNT is an assay detecting NAbs against SARS-CoV-2 that blocks the interaction between the RBD of the viral spike protein with the ACE2 cell surface receptor.^6^ sVNT was performed using a commercial kit (GenScript, Piscataway, NJ) following the manufacturer’s instruction. Briefly, diluted serum samples (or negative and positive controls) were 1:1 mixed with the diluted HRP-RBD (the horseradish peroxidase-conjugated recombinant SARS-CoV-2 RBD fragment) solution, and incubated at 37 °C for 30 min. Add 100 μl each of the mixture to a well in a 96-well plate precoated with human ACE2 receptor protein, and incubate at 37 °C for 15 min. Wash the plate with 260 μl of 1× Wash Solution for four times, add 100 μl of TMB Solution to each well, and incubate the plate in the dark at 25 °C for 15 min. Add 50 μl of Stop Solution to each well to quench the reaction, read the absorbance at 450 nm in a microtiter plate reader immediately. The neutralization rate (NR) was calculated as $\left( {1 - \frac{{OD_{450}\,of\,sample}}{{OD_{450}\,of\,negative\,control}}}\right) \times 100\%$. A NR value of ≥20% at 1:20 sample dilution was regarded positive as suggested by the kit’s manufacturer. IC_50_ of surrogate virus neutralization was calculated from NR values of serially diluted samples using the 4-parameter logistic model.^14^

### SARS-CoV-2 FRNT and calculation of IC_50_

SARS-CoV-2 FRNT was performed in a certified biosafety level 3 lab as previously described.^13^ Fifty-μl serum samples were serially (1:4) diluted, mixed with 50 μl of SARS-CoV-2 (100 focus-forming unit, FFU) in 96-well microwell plates, and incubated for 1 h at 37 °C. Mixtures were then transferred to 96-well plates seeded with Vero E6 cells (ATCC, Manassas, VA) for 1 h at 37 °C. Inoculums were then removed before adding the overlay media (100 μl MEM containing 1.2% carboxymethylcellulose, CMC). The plates were then incubated at 37 °C for 24 h. Overlays were removed and cells were fixed with 4% paraformaldehyde solution for 30 min. Cells were permeabilized with 0.2% Triton X-100 and incubated with cross-reactive rabbit anti-SARS-CoV-N IgG (Cat: 40143-R001, Sino Biological, Inc, Beijing) for 1 h at room temperature before adding HRP-conjugated goat anti-rabbit IgG(H + L) antibody (1:4000 dilution) (Code: 111-035-144, Jackson ImmunoResearch, West Grove, PA). Cells were further incubated at room temperature. The reactions were developed with KPL TrueBlue Peroxidase substrates (Seracare Life Sciences Inc., Milford, MA). The numbers of SARS-CoV-2 foci were calculated using an EliSpot reader (Cellular Technology Ltd., Shaker Heights, OH). IC_50_ of virus neutralization was calculated using the 4-parameter logistic model.

### Measurement of total binding IgG and IgM against SARS-CoV-2

The total binding IgG and IgM antibodies against SARS-CoV-2 were measured using a commercially available Magnetic Chemiluminescence Enzyme Immunoassay kit (Bioscience, Chongqing, China) according to the manufacturer’s instructions. Antibody levels were expressed as the ratio of the chemiluminescence signal over the cutoff value (S/CO). A S/CO value of >1.0 for either IgG or IgM was regarded as positive.

### Statistical analysis

Continuous variables were presented as mean±sd or median (25^th^, 75^th^ percentile), and categorical variables as counts and percentages.

In validation of sVNT, we estimated Spearman’s correlation among IC_50_ titers of FRNT and sVNT, sVNT *NR*_20_, and total binding IgG against SARS-CoV-2. Since all these assays are subject to measurement error, we performed Deming regression with constant variance to assess the linear relationship among log FRNT titers, log sVNT titers, logit *NR*_20_, and log IgG. In the analyses of time to seroconversion for NAbs, IgG, and IgM, we treated time of seroconversion as interval-censored and used Turnbull’s method to calculate the survival curve and obtained the bootstrapped 95% confidence interval for median time to seroconversion.

We defined the peak of the antibodies as the highest level observed between 21^st^ and 60^th^ day post onset. We used linear mixed-effect models with random intercept and random slope to estimate the slope of decline in the titers of NAbs, IgG, and IgM, using titer data on and after the peak. We explored the nonlinearity of the decline rate by adding a quadratic term. For antibodies with nonlinear declining rate, we performed two-segment piecewise linear regression with mixed-effect model to estimate the location of the knot and the segment-specific slopes of decline. We chose the location of the knot that maximized the likelihood of the model. We also evaluated possible modifications of the decline slope by age (>60 or ≤60 years), gender, and peak level (by median of the peak) by adding interaction terms in the model. Decay half-time was calculated as log(0.5)/slope.

To identify clinical correlates that were associated with the peak titer of NAbs (logit *NR*_20_), we used a stepwise linear regression model with Bayesian Information Criteria (BIC) to select variables from a pool of 57 demographic and clinical features, including age, gender, severity and duration of COVID-19 (days from onset to the earliest-time RT-PCR testing turning from positive to negative), presence of symptoms, imaging signs of lung infection, comorbidities such as hypertension, diabetes, and coronary heart disease, complete blood count, and common blood laboratory chemistry tests such as glucose, lipids, electrolytes, liver and kidney functions, and albumin and globulin. A complete list of the variables could be found in Supplementary List 1.

All statistical analyses were performed on R platform (version 3.6.1)

## Supplementary information

Supplemental material

## Data Availability

Data, the statistical code, and technical processes are available from the corresponding author at ffhouguangzhou@163.com.

## Transparency

The paper’s guarantor (FFH) affirms that the paper is an honest, accurate, and transparent account of the study being reported, that no important aspects of the study have been omitted, and any discrepancies from the study as planned have been explained.
