# Supplementary material for: Dynamics of neutralizing antibody responses to SARS-CoV-2 in patients with COVID-19: an observational study
Source: Signal Transduct Target Ther. 2021 May 18;6:197. doi: 10.1038/s41392-021-00611-6 (PMC8129700; doi:10.1038/s41392-021-00611-6)
Supplement: Supplementary file 1 — Supplemental material [file 41392_2021_611_MOESM1_ESM.docx]

Supplementary Materials for

Dynamics of Neutralizing Antibody Responses to SARS-CoV-2 in Patients with COVID-19: An Observational Study

Xin Xu^1#^, Sheng Nie^1#^, Yanqun Wang^2#^, Quanxin Long^3#^, Hong Zhu^1#^, Xiaoyong Zhang^1^, Jian Sun^1^, Qinglang Zeng^4^, Jincun Zhao^2^, Li Liu^1^, Ling Li^5^, Ailong Huang^3^, Jinlin Hou^1*^, Fan Fan Hou^1*^

Correspondence to: [ffhouguangzhou@163.com](mailto:ffhouguangzhou@163.com) or [jlhousmu@163.com](mailto:jlhousmu@163.com)

^1^ State Key Laboratory of Organ Failure Research, National Clinical Research Center for Kidney Disease, Guangdong Provincial Clinical Research Center for Kidney Disease, Nanfang Hospital, Southern Medical University, Guangzhou, China

^2^State Key Laboratory of Respiratory Disease, National Clinical Research Center for Respiratory Disease, Guangzhou Institute of Respiratory Health, the First Affiliated Hospital of Guangzhou Medical University, Guangzhou, China.

^3^Key Laboratory of Molecular Biology on Infectious Diseases, Ministry of Education; Chongqing Medical University, Chongqing, China

^4^The People`s Hospital of Honghu, Honghu, China

^5^Kingmed Medical Laboratory, Guangzhou, China

**This PDF file includes:**

Materials and Methods

Supplementary Text

Figures. S1 to S6

Tables S1

List S1

Figure. S1.

**Flowchart of sample selection.**

Figure. S2.

**The dose response curves of neutralization rate and dilution of sera.** Each curve represents one serum specimen.

Figure. S3.

**Cumulative rate of seroconversion.** In the analysis, time of seroconversion was treated as interval censored and Turnbull’s method was used to estimate the survival curve.

Figure. S4.

**Dynamics of IgG and IgM response to SARS-CoV-2.** The positive rate of IgG and IgM stratified by time post onset. A test with a S/CO value of >1 was regarded as positive. (B) Boxplot of levels of IgG and IgM stratified by time post onset. (C) –(D) Decline of IgG and IgM over time. Each gray line represents the post-peak trajectory of a patient. The blue line is the regression line of the trajectories. The estimated slope was -0.073 (95% CI: -0.082 to -0.064) and -0.163 (95% CI: -0.180 to -0.147) per 10-days for log IgG and log IgM, respectively.

Figure. S5.

Histogram of titers of neutralizing antibodies by FRNT in 197 convalescent sera six months post onset.

Figure. S6.

**Relationship between clinical correlates and peak titer of NAbs.** (A) +/- symptom/imaging denotes presence or absence of symptoms or imaging signs of lung infection, respectively. P-value was calculated by ANOVA test. (B)-(D) Duration of COVID-19 was defined as days from onset to viral shedding (indicated by RT-PCR test turning negative). Blue lines and shaded areas are regression lines and corresponding confidence intervals. Pearson’s correlation coefficients (r) between the clinical feature and peak titer and the two-tailed p-values for testing r=0 are given at the bottom of the plots.

Table S1.

Characters of patients in the analysis of clinical correlates to peak logit NR_20_

| **Variables** | **Peak analysis*  n=328** |
| --- | --- |
| Age, yr | 49.9 ± 14.5 |
| Age, N (%) |  |
| 18 ~ 40 yr | 88 (26.8%) |
| 41 ~ 60 yr | 159 (48.5%) |
| 61 ~ 90 yr | 81 (24.7%) |
| Gender, N (%) |  |
| female | 155 (47.3%) |
| male | 173 (52.7%) |
| Severity, N (%) |  |
| mild | 304 (92.7%) |
| severe | 24 (7.3%) |
| Hypertension, N (%) |  |
| no | 195 (59.5%) |
| yes | 133 (40.5%) |
| Diabetes, N (%) |  |
| no | 283 (86.3%) |
| yes | 45 (13.7%) |
| Coronary heart disease, N (%) |  |
| no | 316 (96.3%) |
| yes | 12 (3.7%) |
| Imaging of lung infection, N (%) |  |
| no | 41 (12.5%) |
| yes | 287 (87.5%) |
| Length of COVID-19^#^, day | 26.8 ± 12.6 |
| Glucose, mmol/L | 6.5 ± 3.0 |
| Total Cholesterol, mmol/L | 4.7 ± 1.2 |
| Albumin, g/L | 40.1 ± 5.7 |
| Globulin, g/L | 27.6 ± 4.7 |
| Leukocyte, 10^9^/L | 5.87 ± 2.52 |
| Lymphocyte, 10^9^/L | 1.53 ± 0.68 |
| eGFR, ml/min/1.73m^2^ | 101 ± 21.7 |

* Data are mean ± sd or N (%)

# defined as days from onset of symptoms to virus shedding (RT-PCR test turning from positive to negative)

List S1.

List of variables used for stepwise regression analysis of peak NAb level.

Dichotomous variables: gender, severity, symptomatic, presence of imaging sign of lung infection, history of hypertension, diabetes, coronary heart disease.

Quantitative variables: age, COVID-19 duration, blood glucose, total cholesterol, HDL, LDL, triglyceride, eGFR, BUN, uric acid, alkaline phosphatase, aspartate aminotransferase, glutamine glutamyl transferase, alanine aminotransferase, total, direct and indirect bilirubin, albumin, globulin, total protein, A/G ratio, Na^+^, K^+^, Cl^-^, Ca^++^, carbon dioxide combining power, RBC, WBC, platelet, hemoglobin, mean hemoglobin concentration, mean platelet volume, eosinophil count, basophil count, neutrophil count, monocyte count, lymphocyte count, nucleated RBC, eosinophil%, basophil%, neutrophil%, monocyte%, lymphocyte%, nucleated RBC%, platelet distribution width, plateletcrit, platelet-large cell ratio, RBC distribution width SD, RBC distribution width CV.
